# Wild barley introgression lines revealed novel QTL alleles for root and related shoot traits in the cultivated barley (*Hordeum vulgare* L.)

**DOI:** 10.1186/s12863-014-0107-6

**Published:** 2014-10-07

**Authors:** Ali Ahmad Naz, Md Arifuzzaman, Shumaila Muzammil, Klaus Pillen, Jens Léon

**Affiliations:** Institute of Crop Science and Resource Conservation, Crop Genetics and Biotechnology Unit, University of Bonn, Katzenburgweg 5, Bonn, 53115 Germany; Institute of Agricultural and Nutritional Sciences, Chair of Plant Breeding, Martin-Luther-University Halle-Wittenberg, Betty-Heimann-Str. 3, Halle, 06120 Germany

**Keywords:** QTL analysis, Introgression lines, Root traits, Drought stress, Wild barley

## Abstract

**Background:**

Root is the prime organ that sucks water and nutrients from deep layer of soil. Wild barley diversity exhibits remarkable variation in root system architecture that seems crucial in its adaptation to abiotic stresses like drought. In the present study, we performed quantitative trait locus (QTL) mapping of root and related shoot traits under control and drought conditions using a population of wild barley introgression lines (ILs). This population (S42IL) comprising of genome-wide introgressions of wild barley accession ISR42-8 in the cultivar Scarlett background. Here, we aimed to detect novel QTL alleles for improved root and related shoot features and to introduce them in modern cultivars.

**Results:**

The cultivar Scarlett and wild barley accession ISR42-8 revealed significant variation of root and related shoot traits. ISR42-8 showed a higher performance in root system attributes like root dry weight (RDW), root volume (RV), root length (RL) and tiller number per plant (TIL) than Scarlett. Whereas, Scarlett exhibited erect type growth habit (GH) as compared to spreading growth habit in ISR42-8. The S42IL population revealed significant and wide range of variation for the investigated traits. Strong positive correlations were found among the root related traits whereas GH revealed negative correlation with root and shoot traits. The trait-wise comparison of phenotypic data with the ILs genetic map revealed six, eight, five, five and four QTL for RL, RDW, RV, TIL and GH, respectively. These QTL were linked to one or several traits simultaneously and localized to 15 regions across all chromosomes. Among these, beneficial QTL alleles of wild origin for RL, RDW, RV, TIL and GH, have been fixed in the cultivar Scarlett background.

**Conclusions:**

The present study revealed 15 chromosomal regions where the exotic QTL alleles showed improvement for root and related shoot traits. These data suggest that wild barley accession ISR42-8 bears alleles different from those of Scarlett. Hence, the utility of genome-wide wild barley introgression lines is desirable to test the performance of individual exotic alleles in the elite gene pool as well as to transfer them in the cultivated germplasm.

## Background

Drought is the most common abiotic stress that causes around 70% yield losses in crops in conjunction with heat and salinity [1,2]. These losses are one of the reasons behind the sufferings of around one billion people living in chronic hunger world-wide [3]. The morphological traits related to water-use efficiency appear to play a fundamental role in drought tolerance/avoidance in crop plants [4,5]. Therefore, dissecting the genetic basis of such traits can offer potential leads for selection in plant breeding to develop drought resilient cultivars that may help to bridge the gap of food shortage in the world [6,7].

Roots are the first plant organ that perceives water deficit signals and transduce them to shoot which in turn cuts down its water budget via stomatal closure and the cessation of its development and growth [8]. Hence, a long period of drought leads to dramatic losses in shoot biomass and crop yield or under extreme drought scenario inability of crop plants to survive. An extensive rooting to access water from deeper soils layers is prevalent in land plants adapted to water deficit or rain-fed conditions. A vigorous root system (depth, orientation and branching) leads to a greater contact between roots and soil which in turn enhances the uptake of water and nutrients, favorable gas-exchange and carbon assimilation [9]. Moreover, root can suck water even from the drier layer of soil, thus bear the ability to differentiate and grow under extreme drought conditions. Therefore, an extensive rooting is desirable trait in crops. Recently, Uga et al. [10] reported that positional cloning of QTL underlying *DEEPER ROOTING 1* (*Dro1*) gene in rice by using a cross between an upland deep rooting rice cultivar Kinandang Patong with a lowland shallow rooting cultivar IR64. They developed near isogenic line (NIL) containing *Dro1* in the IR64 background via marker assisted selection. *Dro1*-NIL demonstrated significant increase in shoot biomass, yield and drought stress avoidance as compared to control genotype IR64 suggesting that the alteration of root system architecture improves yield and drought avoidance in rice.

Barley (*Hordeum vulgare* L.) root system is composed of two distinct components: (1) the seminal roots that originate from primordia in embryo, and (2) the nodal roots that arise from basal nodes of the main shoot and tillers [11,12]. The seminal roots develop first and function until the nodal roots become established. Both of these roots eventually initiate lateral roots (secondary and tertiary roots) on which water and nutrient absorbing root hairs are developed [13]. It has been reported that the number of roots in a plant is closely related to the tiller number per plant [14,15]. For example, Anderson-Taylor and Marshall [16] reported seminal and nodal roots of spring barley (*H. distichum* L.) comprised of 40 and 60 percent, respectively of total roots and nodal root dry weight was correlated with primary tillers. Chloupek et al. [17] studied root system size (RSS) in barley (*H. vulgare* L.) under drought conditions and observed significant positive correlations between RSS and grain yield. Tiller number per plant is a major determinant of yield in crops like barley. Therefore, proper dissection and understanding of root attributes facilitating water uptake under drought can help breeders to elucidate essential traits for drought tolerance. Barley reveals great diversity in its root pattern, size and architecture. In general, wild barley accessions showed much higher variation due to its diverse ecological adaptation [18,19]. Tyagi et al. [20] evaluated drought stress tolerance in wild barley accessions of different origins and reported the highest level of drought stress tolerance in wild barley accessions collected from Israel and Jordan. It highlights the existence of valuable diversity within the wild barley gene pool. Therefore, it is necessary to dissect the genetic basis of this natural diversity to identify vital genes for improved root attributes and drought stress tolerance to introduce them in the modern cultivars [21].

Tanksley and Nelson [22] devised advanced backcross-quantitative trait locus (AB-QTL) strategy that allows a targeted detection and transfer of the favorable exotic alleles into elite breeding material. Several AB-QTL studies were performed in barley for morphological and agronomic traits, malting quality, disease resistance and tolerance to drought stress [23-26]. In a similar approach, Zamir [27] developed genome-wide introgression lines (ILs) libraries where marker-defined genomic regions taken from wild species were introgressed onto the background of elite crop lines. This material allows a straightforward comparison of ILs with the elite recurrent parent to dissect the effects of wild introgressions in a near isogenic background. Such genetic background is essential for detection, validation and positional cloning of QTL. Till now, QTL detection studies in relation to root related drought stress using ILs or NILs were conducted in rice [28], wheat [29], maize [30], tomato [31] and chickpea [32] etc. In barley, Schmalenbach et al. [33] developed an introgression line library S42IL encompassing the wild barley (ISR42-8) introgressions in the background of cultivar Scarlett. Naz et al. [13] identified and validated QTL on chromosome 5H for root-related traits using two ILs among this population. Hoffmann et al. [34] detected nitrogen deficiency QTL using 42 ILs of this population growing in hydroponic system. However, identification of genome-wide QTL for root and related shoot traits using complete set of the S42IL population under control and drought stress conditions is still missing. Therefore, in the present study we aimed to execute genome-wide exploration of QTL related to root and shoot traits using a set of 72 ILs of the S42IL population under control and drought stress conditions. The final goal was to identify beneficial QTL alleles of wild origin for root and related shoot parameters and to utilize them for breeding as well as for positional cloning of the underlying genes.

## Methods

### Plant material

A wild barley introgression library consisting of 72 lines was used in the present study. The introgression lines (ILs) were developed from an initial cross between the German spring cultivar Scarlett (*Hordeum vulgare* ssp. *vulgare*) and the wild barley accession ISR42-8 (*H. vulgare* ssp. *spontaneum*) from Israel. The resultant F1 cross was backcrossed two times with recurrent parent Scarlett and 301 BC_2_DH population was produced which is known as S42 population. From this population, 40 lines were selected through marker assisted selection and backcrosssed again with Scarlett. After several rounds of selfing and marker-assisted selection, BC_3_S_6_ population was produced. This population was designated as S42IL and the details of its development and marker genotyping can be found in Schmalenbach et al. [33].

### Phenotypic evaluation of traits

The experiment was arranged in a split-plot design with three replications in 2012 and four replications in 2013. The treatments (control and drought) were assigned to the sub-plots in which lines were assigned randomly. For this, four seeds of individual S42IL were sown in plastic pots (22 × 22 × 26 cm) containing a mixture of top soil, silica sand, milled lava and peat dust (Terrasoil®, Cordel & Sohn, Salm, Germany). Water supply was done with a drip irrigation system (Netafilm, Adelaide, Australia) by watering pots three times per day. Echo2 sensors (Decagon Dev., Pullman WA, USA) were used to determine the volumetric moisture content (VMC) digitally with the frequency domain technique. The drought stress treatment was carried out 30 days after sowing (DAS) by eliminating the water supply completely at plant development stage BBCH 29–31 [35]. The plants were kept under stress for 26 days till VMC reached the maximum drought stress threshold near to wilting point (VMC near to 0%). The control block was kept under continuous supply of irrigation. The mean average temperature during the experimental period was 18.2°C in 2012 and 14.2°C in 2013. The relative humidity in 2012 and 2013 was 59.5% and 64.1%, respectively.

Five root and related shoot traits which were evaluated as follows:

- Root length (RL): After manual root washing, RL was measured from the stem base to the root tip by spreading the complete root on a ruler in centimetres (cm).
- Root dry weight (RDW): Roots were dried in the oven at 50°C for seven days and weighed in grams (g).
- Root volume (RV): Measured in cubic centimetres (cm^3^) by calculating the volume differences between before and after immersing the total roots in a 500 ml measuring cylinder containing water.
- Tiller number per plant (TIL): Before harvesting, total number of tillers were counted in each plant.
- Growth habit (GH): Plants were scored from 1 to 5 considering spreading growth type (1) to erect growth type (5).

### Genotypic data

Genetic map of the S42IL population was achieved using 1536-SNP barley BOPA1 set according to Schmalenbach et al. [36].

### Statistical analyses

Statistical analyses were carried out using the software package SAS Enterprise 9.2 [37]. The variance analysis of S42ILs was estimated with PROC GLM procedure as follows:

1 $$ {Y}_{ijk}=\mu + {G}_i + {T}_j + {Y}_k + {G}_i \times {T}_j + {T}_j\left({Y}_k\right) + {\varepsilon}_{ijk} $$

where *μ* is the general mean, *G*_*i*_ the fixed effect of _*i-*_th genotype, *T*_*j*_ the fixed effect of _*j-*_th treatment, *Y*_*k*_ the random effect of _*k-*_th year, *G*_*i*_ *× T*_*j*_ the fixed interaction effect of the _*i-*_th genotype with _*j-*_th treatment, *T*_*j*_(*Y*_*k*_) the random effect of the _*k-*_th year.

The PROC VARCOMP in SAS was used to measure the variance components of genotype (V_*G*_), genotype by treatment (*V*_*G* × *T*_), genotype by year (*V*_*G* × *Y*_) and experimental error (*V*_*E*_). Coefficients of broad-sense heritability (h^2^) were performed for five studied traits across all the treatments according to Holland et al. [38]:

2 $$ {\mathrm{h}}^2=\frac{{\mathrm{V}}_G}{{\mathrm{V}}_G+\frac{{\mathrm{V}}_{G\times T}}{t}+\frac{{\mathrm{V}}_{G\times Y}}{y}+\frac{{\mathrm{V}}_E}{tyr}} $$

where *t*, *y* and *r* are the number of treatments (*t* = 2), number of years (*y* = 2) and the average number of replications (*r* = 3.5), respectively.

Least square means (Lsmeans) were calculated with GLM procedure considering all replications and years separately for both control and drought conditions. Genetic correlation coefficients (r) between traits were estimated using Lsmeans of 72 S42ILs with CORR procedure in SAS.

### QTL detection

For QTL detection, the post-hoc Dunnet test was applied to see the significant differences between the individual introgression lines of the S42IL population and Scarlett [39]. The QTL detection was assumed if the individual introgression lines revealed significant difference to Scarlett across both treatments. Later, putative QTL regions were refined by comparing the common overlapping wild introgressions among the ILs having QTL alleles of wild origin as well as by comparing wild introgression across chromosomal regions having no QTL effect.

The quantification of QTL effects was made by calculating the relative performance (RP) of particular S42IL introgression line bearing the QTL in comparison to control parent Scarlett by the following formula:

3 $$ RP(S42IL)=\frac{Lsmeans(S42IL)- Lsmeans(Scarlett)}{Lsmeans(Scarlett)}\times 100 $$

## Results

### Variance analyses

The variance analyses revealed highly significant variation among the genotypes for root length (RL), root dry weight (RDW), root volume (RV), tiller number per plant (TIL) and growth habit (GH, Table 1). The treatment effect was significant for RDW, RV and TIL, whereas years presented highly significant variations for most traits except GH. The effects for genotype by treatment and treatment by year interactions were significant for RL, RDW, RV and TIL. Non-significant differences were observed between replications across years for all traits except TIL. The highest heritability (h^2^) was found for GH (0.99) whereas RL, RDW, RV, TIL revealed 0.72, 0.56, 0.64, 0.76 h^2^, respectively.

**Table 1:** **Variance analyses of five investigated traits among 33 common S42ILs and parents across the years 2012 and 2013 under control and drought treatments**

**Trait** ^**a**^	**SOV** ^**b**^	**DF** ^**c**^	**MS** ^**d**^	**F value**	**P value** ^**e**^	**h** ^**2f**^
RL	Genotype	34	497.6	11.1	<0.001	0.72
	Treatment	1	147.8	3.3	ns	
	Genotype × Treatment	34	110.5	2.5	<0.001	
	Year	1	1628.4	36.2	<0.001	
	Treatment × Year	1	348.5	7.7	<0.01	
	Replication (Year)	5	84.0	1.9	ns	
RDW	Genotype	34	26.5	15.2	<0.001	0.56
	Treatment	1	83.1	47.6	<0.001	
	Genotype × Treatment	34	7.7	4.4	<0.001	
	Year	1	120.2	68.8	<0.001	
	Treatment × Year	1	17.0	9.7	<0.01	
	Replication (Year)	5	1.2	0.7	ns	
RV	Genotype	34	1999.5	15.8	<0.001	0.64
	Treatment	1	6556.4	51.8	<0.001	
	Genotype × Treatment	34	452.4	3.6	<0.001	
	Year	1	17003.4	134.4	<0.001	
	Treatment × Year	1	2321.9	18.4	<0.001	
	Replication (Year)	5	67.4	0.5	ns	
TIL	Genotype	34	22.7	12.5	<0.001	0.76
	Treatment	1	664.1	365.3	<0.001	
	Genotype × Treatment	34	4.4	2.4	<0.001	
	Year	1	16.6	9.1	<0.01	
	Treatment × Year	1	136.0	74.7	<0.001	
	Replication (Year)	5	25.1	13.8	<0.001	
GH	Genotype	34	15.3	536.4	<0.001	0.99
	Treatment	1	0.1	0.7	ns	
	Genotype × Treatment	34	0.1	0.4	ns	
	Year	1	0.1	0.7	ns	
	Treatment × Year	1	0.1	0.2	ns	
	Replication (Year)	5	0.1	1.3	ns	

^a^Trait: RL = Root length, RDW = Root dry weight, RV = Root volume, TIL = Tiller number per plant, GH = Growth habit.

^b^Sources of variation.

^c^Degrees of freedom.

^d^Mean sum of square.

^e^P-value indicates the level of significance at <0.05, <0.01 and <0.001; ns: not significant.

^f^Heritability.

### Phenotypic characterization

Trait-wise means comparison among Scarlett, ISR42-8 and the S42IL population is presented in Table 2. S42ILs, Scarlett and ISR42-8 revealed significant variation for most of the traits under control and drought conditions. Under control conditions, the maximum RL 84.0 cm was observed in ISR42-8 whereas the S42IL population accounted for maximum RL (81.0 cm) which was higher than ISR42-8 (64.0 cm) and Scarlett (44.0 cm) under drought stress conditions. ISR42-8 revealed maximum RDW under both control (27.1 g) and drought (10.7 g) conditions. The S42IL population accounted for remarkable variation in RDW that ranged from 1.7 to 11.5 g under control and 2.0 to 8.8 g under drought stress conditions. RV showed similar trend of variation like RDW. TIL was higher in ISR42-8 in both control and drought conditions compared to Scarlett. However, the S42IL population revealed maximum TIL 20.0 under control conditions. Under drought, the S42IL population revealed maximum TIL 13.3 which was almost similar to TIL (14.0) in ISR42-8. For GH, we used a scale from 1 (spreading GH) to 5 (erect type GH). ISR42-8 showed spreading GH (1) under control and drought stress conditions where Scarlett was found as erect type (score 5). S42ILs revealed a range of GH (score 2 to 5) under both control and drought conditions.

**Table 2:** **Mean comparison of root and related shoot traits among the S42IL population, Scarlett and ISR42-8 under control and drought stress conditions**

**Trait** ^**a**^	**Genotype**	**Mean** ^**b**^ ± **SE** ^**c**^		**Minimum**		**Maximum**	
		**Control**	**Drought**	**Control**	**Drought**	**Control**	**Drought**
RL	S42IL	46.4^b^ ± 0.5	46.0^b^ ± 0.5	25.0	18.0	82.0	81.0
	ISR42-8	64.9^a^ ± 4.1	56.0^a^ ± 2.2	52.0	48.0	84.0	64.0
	Scarlett	42.4^c^ ± 1.1	37.4^c^ ± 1.3	37.0	34.0	45.0	44.0
RDW	S42IL	4.6^b^ ± 0.1	4.1^b^ ± 0.1	1.7	2.0	11.5	8.8
	ISR42-8	14.9^a^ ± 2.7	6.9^a^ ± 1.0	7.0	4.5	27.1	10.7
	Scarlett	3.4^c^ ± 0.2	3.1^c^ ± 0.1	2.7	2.8	3.8	3.4
RV	S42IL	43.9^b^ ± 0.8	38.3^b^ ± 0.5	10.0	14.0	120.0	80.0
	ISR42-8	130.3^a^ ± 18.9	67.3^a^ ± 10.5	80.0	43.0	210.0	115.0
	Scarlett	34.3^c^ ± 2.3	32.3^c^ ± 0.9	25.0	30.0	40.0	35.0
TIL	S42IL	10.1^b^ ± 0.1	7.8^b^ ± 0.2	6.5	3.8	20.0	13.3
	ISR42-8	16.5^a^ ± 0.4	11.3^a^ ± 0.8	14.3	8.5	17.8	14.0
	Scarlett	8.7^c^ ± 0.4	6.9^b^ ± 0.7	6.8	4.8	9.8	9.3
GH	S42IL	4.5^b^ ± 0.1	4.5^b^ ± 0.2	2	2	5	5
	ISR42-8	1^a^ ± 0.0	1^a^ ± 0.0	1	1	1	1
	Scarlett	5^c^ ± 0.0	5^c^ ± 0.0	5	5	5	5

^a^Trait: RL = Root length (cm), RDW = Root dry weight (g), RV = Root volume (cm^3^), TIL = Tiller number per plant, GH = Growth habit.

^b^The means of S42ILs, ISR42-8 and Scarlett were calculated as an average of the phenotypic data for each trait across 2012 and 2013 for each treatment separately. Statistical differences across means are indicated with letters a, b and c.

^c^Standard error.

### Genetic correlations

The correlation coefficients among the traits are presented in Table 3. Under control conditions, RL revealed highly significant and positive correlations with RDW (0.65) and RV (0.68). A moderate positive correlation of RL was found with TIL (0.40) and a negative correlation with GH (−0.35). The strongest positive correlation was detected between RDW and RV (0.93). RV was moderately correlated with TIL (0.51) and GH (−0.46). TIL revealed highly significant and negative correlation with GH (−0.68). Under drought conditions, RL exhibited strong and positive correlation with RDW (0.60) and RV (0.56) but weak correlation with TIL and GH. Again, RDW showed the strongest correlation with RV (0.79). TIL showed strong and negative correlation with GH (0.62).

**Table 3:** **Pearson correlation coefficients (r) among root and related shoot traits under control and drought stress conditions**

**Treatment**	**Trait** ^**a**^	**RL**	**RDW**	**RV**	**TIL**	**GH**
Control	RL	1				
	RDW	0.65***	1			
	RV	0.68***	0.93***	1		
	TIL	0.40**	0.57***	0.51***	1	
	GH	−0.35**	−0.50***	−0.46***	−0.68**	1
Drought	RL	1				
	RDW	0.60***	1			
	RV	0.56***	0.79***	1		
	TIL	0.23*	0.27*	0.28*	1	
	GH	−0.27*	−0.35**	−0.41***	−0.62***	1

^a^Trait: RL = Root length, RDW = Root dry weight, RV = Root volume, TIL = Tiller number per plant, GH = Growth habit.

*, **, ***indicates significant at <0.05, <0.01 and <0.001 levels of probability, respectively.

### QTL detection

**Figure 1:**
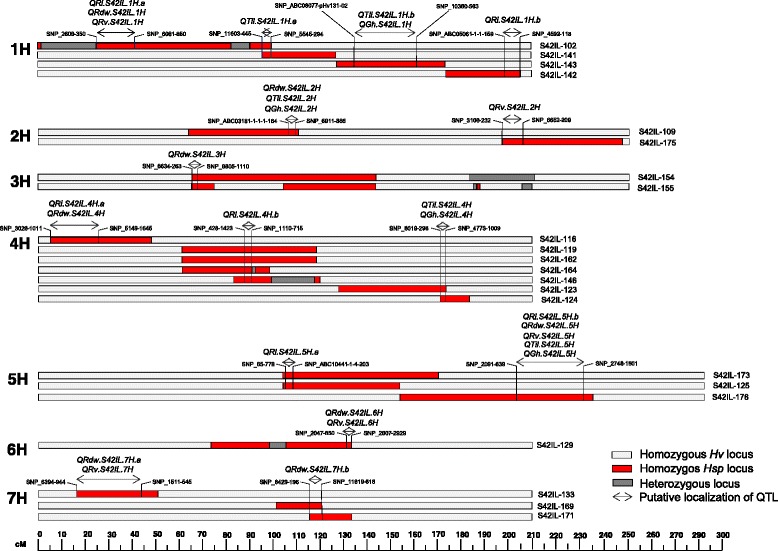
**Chromosomal localization of QTL effects for root and related shoot traits.** The cM positions of the SNP loci are indicated with ruler at the bottom according to Schmalenbach et al. [36]. The QTL regions are narrowed by comparing the common overlapping introgression across the S42IL population as well as by comparing QTL bearing wild introgression with the chromosomal regions having no QTL.

**Figure 2:**
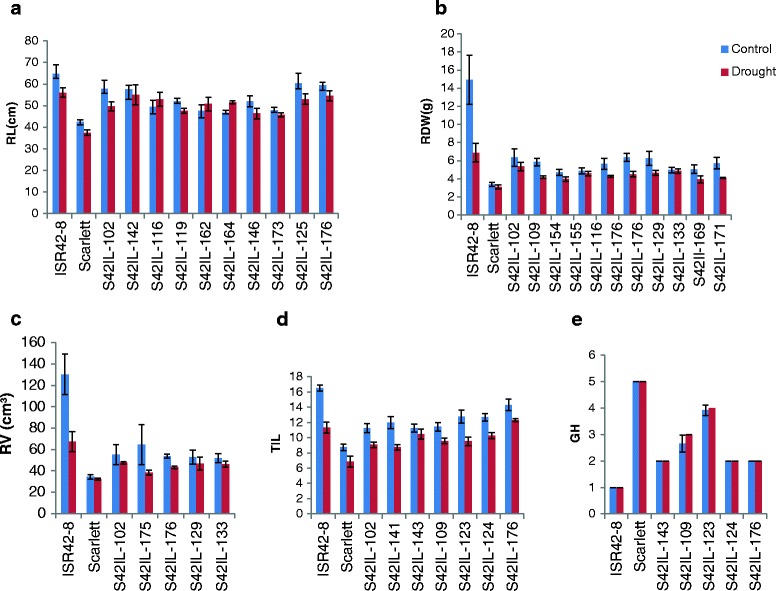
**Quantification of QTL alleles for root length (a), root dry weight (b), root volume (c), tiller number per plant (d) and growth habit (e).** The position of related QTL can be found in Table 4.

**Figure 3:**
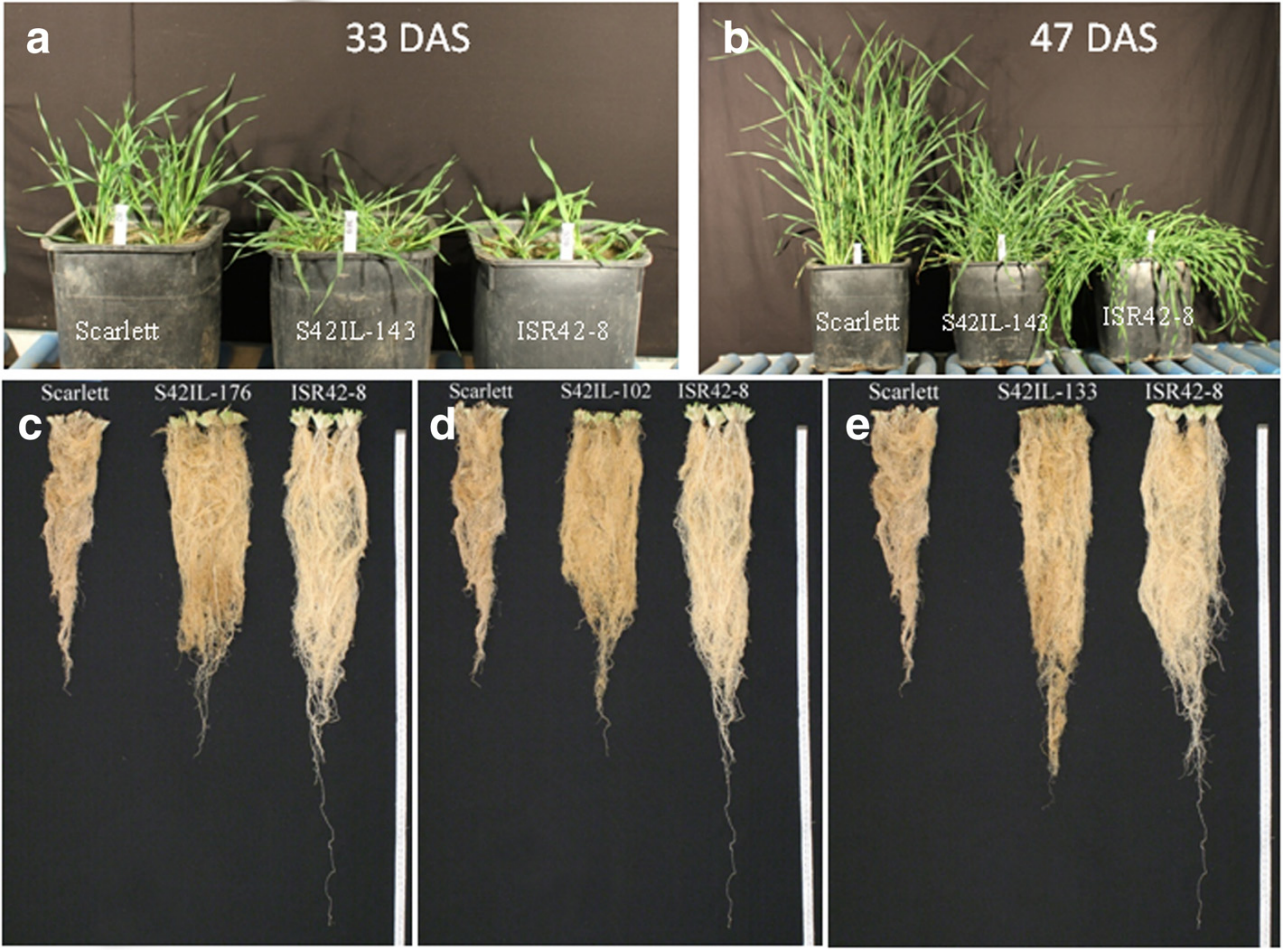
**Comparison of selected introgression lines (ILs) with Scarlett and ISR42-8. (a-b)** comparison of S42IL-143 revealing QTL alleles for GH and TIL with Scarlett and ISR42-8 at 33 and 47 days after sowing (DAS). (**c** to **e**) root variation among ILs S42IL-176 **(c)**, S42IL-102 **(d)** and S42IL-133 **(e)** in comparison to Scarlett and ISR42-8.

Altogether, 28 significant QTL effects were identified for five root and related shoot traits (Table 4). These QTL effects are localized to 15 chromosomal regions across all chromosomes (Figure 1). The comparison and quantification of ILs having agronomically beneficial QTL allele with Scarlett and ISR42-8 are presented in Figures 2 and 3.

**Table 4:** **List of QTL effects for root and related shoot traits detected in the S42IL population**

**Trait** ^**a**^	**QTL name**	**Chr** ^**b**^	**Introgression (cM)**	**S42IL**	**Lsmeans S42IL** ^**c**^	**Lsmeans Scarlett** ^**d**^	**RP(IL)** ^**e**^ **(%)**
RL	*QRl.S42IL.1H.a*	1H	24.17-40.51	S42IL-102	54.1	40.2	34.5
	*QRl.S42IL.1H.b*	1H	199.04-205.07	S42IL-142	56.3		40.1
	*QRl.S42IL.4H.a*	4H	5.42-26.58	S42IL-116	51.4		28.0
	*QRl.S42IL.4H.b*	4H	89.78-91.93	S42IL-119	50.1		24.6
				S42IL-162	50.4		25.3
				S42IL-164	49.1		22.2
				S42IL-146	55.6		38.3
	*QRl.S42IL.5H.a*	5H	105.91-109.27	S42IL-173	46.5		15.7
				S42IL-125	56.9		41.6
	*QRl.S42IL.5H.b*	5H	203.85-231.75	S42IL-176	57.0		41.8
RDW	*QRdw.S42IL.1H*	1H	24.17-40.51	S42IL-102	5.9	3.3	78.8
	*QRdw.S42IL.2H*	2H	107.63-108.71	S42IL-109	5.1		54.5
	*QRdw.S42IL.3H*	3H	64.85-65.96	S42IL-154	4.5		36.3
				S42IL-155	4.8		45.5
	*QRdw.S42IL.4H*	4H	5.42-26.58	S42IL-116	5.1		54.5
	*QRdw.S42IL.5H*	5H	203.85-231.75	S42IL-176	5.4		63.6
	*QRdw.S42IL.6H*	6H	133.29-133.47	S42IL-129	5.5		66.6
	*QRdw.S42IL.7H.a*	7H	17.32-44.83	S42IL-133	5.0		51.5
	*QRdw.S42IL.7H.b*	7H	118.80-120.52	S42IL-169	4.5		36.3
				S42IL-171	4.9		48.5
RV	*QRv.S42IL.1H*	1H	24.17-40.51	S42IL-102	52.4	34.1	53.6
	*QRv.S42IL.2H*	2H	197.39-206.17	S42IL-175	51.4		50.7
	*QRv.S42IL.5H*	5H	203.85-231.75	S42IL-176	48.5		42.2
	*QRv.S42IL.6H*	6H	133.29-133.47	S42IL-129	50.6		48.1
	*QRv.S42IL.7H*	7H	17.32-44.83	S42IL-133	51.1		49.8
TIL	*QTil.S42IL.1H.a*	1H	97.18-98.23	S42IL-102	10.1	7.8	29.5
				S42IL-141	10.3		32.1
	*QTil.S42IL.1H.b*	1H	134.94-161.60	S42IL-143	10.8		38.5
	*QTil.S42IL.2H*	2H	107.63-108.71	S42IL-109	10.5		34.6
	*QTil.S42IL.4H*	4H	171.25-172.32	S42IL-123	10.6		35.9
				S42IL-124	11.4		46.2
	*QTil.S42IL.5H*	5H	203.85-231.75	S42IL-176	13.3		70.5
GH	*QGh.S42IL.1H*	1H	134.94-161.60	S42IL-143	2.0	5.0	−60
	*QGh.S42IL.2H*	2H	63.96-110.84	S42IL-109	3.0		−40
	*QGh.S42IL.4H*	4H	171.25-172.32	S42IL-123	3.9		−28
				S42IL-124	2.0		−60
	*QGh.S42IL.5H*	5H	203.85-231.75	S42IL-176	2.0		−60

^a^Trait: RL = Root length (cm), RDW = Root dry weight (g), RV = Root volume (cm^3^), TIL = Tiller number per plant, GH = Growth habit.

^b^Chromosome number.

^c,d^Least square means of the S42IL and Scarlett, respectively.

^e^Relative trait performance of the S42IL compared to Scarlett, calculated as RP(S42IL) = [Lsmeans(S42IL) – Lsmeans(Scarlett)]/Lsmeans(Scarlett).

#### Root length (RL)

Six putative QTL were detected for RL located on chromosomes 1H, 4H and 5H. The quantification of these QTL was made by comparing the ILs revealing beneficial QTL allele with Scarlett and ISR42-8. The strongest QTL *QRl.S42IL.5H.b* was found in S42IL-176 on chromosome 5H between 203.85 to 231.75 cM which resulted in 41.8% increase in RL as compared to Scarlett. Another QTL for RL was detected on chromosome 5H between 105.91 to 109.27 cM where the IL S42IL-125 accounted for 41.6% increase in trait value. A QTL of similar phenotypic performance was found on the long arm of chromosome 1H which accounting for 40.1% increase in RL. Two QTL were identified on chromosome 4H, one on each arm, short and long. ILs bearing these exotic QTL alleles resulted in a moderate increase in RL ranging from 22.2 to 38.3%.

#### Root dry weight (RDW)

The QTL analysis revealed eight QTL for RDW distributed across chromosomes 1H to 7H. According to the relative performance (RP) of exotic allele, *QRdw.S42IL.1H* exhibited the strongest QTL effect for RDW by S42IL-102 (78.8%) on chromosome 1H. This QTL spans from 24.17 to 40.51 cM on chromosome 1H. The second strongest QTL *QRdw.S42IL.6H* was detected in S42IL-129 on chromosome 6H between 133.29 to 133.47 cM which improve RDW by 66.6%. Two QTL *QRdw.S42IL.7H.a* and *QRdw.S42IL.7H.b* were identified on the short and long arms of chromosome 7H which enhanced RDW by 51.5% and 48.5%, respectively. Likewise, wild introgression on chromosome 2H between 107.63 to 108.71 cM was associated with 54.5% increase in RDW. A common QTL *QRdw.S42IL.3H* was found in S42IL-154 and S42IL-155 on chromosome 3H which revealed 36.3 and 45.5% enhancement of RDW in these ILs, respectively.

#### Root volume (RV)

Five putative QTL for RV were mapped on chromosomes 1H, 2H, 5H, 6H and 7H. These QTL effects revealed preeminence of exotic allele which ranged from 42.2 to 53.6%. All the genotypes showed higher RV in control than in drought conditions. The strongest QTL effect (*QRv.S42IL.1H.a*) was detected on chromosome 1H between 24.17 to 40.51 cM in IL S42IL-102. The second strongest QTL (*QRv.S42IL.2H*) revealed the superior performance of an exotic allele on the long arm of chromosome 2H which accounted for 50.7% increase in RV. *QRv.S42IL.6H* and *QRv.S42IL.7H* on chromosomes 6H and 7H showed 48.1% and 49.8% increase in RV, respectively.

#### Tiller number per plant (TIL)

Five QTL were identified for TIL located on chromosomes 1H, 2H, 4H and 5H. The strongest exotic QTL *QTil.S42IL.5H* was detected on chromosome 5H between 203.85 to 231.75 cM which accounted for 70.5% increase in TIL. S42IL-124 revealed the second strongest QTL *QTil.S42IL.4H* on chromosome 4H (171.25-172.32 cM). The *QTil.S42IL.1H.a* exhibited a moderate increase in the relative performance in ILs S42IL-102 and S42IL-141 by 29.5 and 32.1%, respectively.

#### Growth habit (GH)

Four QTL were found for GH located on chromosomes 1H, 2H, 4H and 5H. S42IL-143, S42IL-124 and S42IL-176 revealed spreading GH (score 2) similar to ISR42-8 and accounted for around 60% decrease in the Scarlett erect type GH. These QTL were linked to wild introgression on chromosome 1H (*QGh.S42IL.1H*), 4H (*QGh.S42IL.4H*) and 5H (*QGh.S42IL.5H*). Another remarkable QTL on chromosome 2H revealed 40% decrease in the erect type GH in IL S42IL-109.

## Discussion

### Phenotypic evaluations

Root architecture takes part in plant adaptation under water scarce conditions [40]. The wild barley accessions bears an inherent ability to develop an extensive and deep rooting that provides an opportunity to utilize their natural diversity to improve root system in modern cultivars. Recently, Naz et al. [13] reported dramatic differences of root system attributes between Scarlett and ISR42-8 while mapping root system variation in two selected introgression lines S42IL-176 and S42IL-126. These results suggest that ISR42-8 may contain additional unique alleles for root system variation. Therefore, in the present study we aimed to execute a whole genome mapping of root system variation using a set of wild barley introgression lines having genome-wide chromosomal segments of ISR42-8 in the Scarlett background. We have found a vigorous root system in wild barley accession ISR42-8 in comparison to Scarlett under control and drought conditions. The S42IL population showed significant variation for RL, RDW, RV, TIL and GH indicating the segregation of selected traits across 72 ILs. This data present a number of phenotypic classes for RL, RDW, RV and TIL among the S42IL population suggesting quantitative inheritance of root attributes and tillering. Hoffmann et al. [34] also found significant variation for RL, RDW and tiller number in a set of 42 IL of the S42IL population across two different levels of nitrogen under hydroponic conditions. Interestingly, mean comparisons revealed superior performance of an introgression line than ISR42-8 for RL under drought stress conditions. This fact leads us to speculate the presence of drought inducible transgressive exotic allele whose expression in the Scarlett background might be advantageous to RL. Likewise, a transgressive effect for TIL was detected under control conditions. These data revealed the presence of valuable alleles in wild barley and suggest their utility in the elite gene pool. Gu et al. [41] revealed transgressive segregation in ILs of rice under drought and well-watered field conditions. Taken together, higher RL and RDW enhanced access to water from deeper layers of soil which putatively enabled the introgression line to maintain more favorable gas-exchange and carbon assimilation levels and mineral nutrient uptake during water stress conditions [42,43].

The magnitude of traits heritability (h^2^) and stability are important criteria for designing plant breeding programs. Traits conferring higher heritability across different environments could easily be selected for breeding. In this study, RL, RDW, RV, TIL and GH showed 0.72, 0.56, 0.64, 0.76 and 0.99 h^2^, respectively across the years 2012 and 2013. This finding indicates that these traits are heritable as well as stable across the years. Hoffmann et al*.* [34] found very strong heritability for RL (0.85) but relatively lower for tiller number (0.31) and RDW (0.32) in hydroponic system under two regimes of nitrogen in 42 ILs of similar population. We also tested the relationship of root and shoot by calculating their correlations among each other. The strongest correlation was found between RDW and RV confirming the logical relationship of root weight and volume. Furthermore, RL also showed significant correlation with RDW and RV suggesting the likelihood of common genetic components influencing these traits simultaneously. Sandhu et al. [44] reported a strong positive correlation among RL, root thickness and RV in two mapping populations of rice. We observed TIL was positively correlated with RL, RDW and RV in both control and drought conditions. ISR42-8 revealed a spreading type GH and delayed flowering which seems the major reason of more TIL and consequently higher values of root traits as barley develops nodal roots from each tiller during its development. These findings are in line with Shin et al. [45] where they found that the total number of nodes per plant determines the total number of roots. Naz et al. [13] also reported strong positive relationship among RV, RDW, RL and change in tiller numbers.

### QTL localization

The localization of root and shoot QTL on the ILs genetic map revealed 15 chromosomal regions affecting one or several traits. These QTL bearing regions were located across chromosomes 1H to 7H. The IL S42IL-102 bore QTL for RL, RDW and RV that revealed the highest effect on RDW by increasing the trait value by 78.8%. This QTL spans between 24.17 to 40.51 cM on chromosome 1H and appears to underlie a major gene for root development that influences multiple root traits like RDW, RV and RL. Sayed [46] reported QTL for RDW on chromosome 1H (position 39 cM) using 301 BC_2_DH lines of which these ILs were derived. Validation of this QTL in different background showed the stability of this exotic QTL allele in the Scarlett background. Similarly, a QTL between 134.94 to 161.60 cM on chromosome 1H that was associated with more TIL and spreading GH in IL S42IL-143 similar to donor parent ISR42-8. This IL showed late flowering (data not shown) because it carried the wild barley version of *HvFT3* allele. Wang et al. [47] showed that wild allele of *HvFT3* is linked to delayed flowering in the background of Scarlett. In barley, delayed flowering promotes vegetative growth, therefore, it is tempting to speculate that the increase in TIL and wild type GH in S42IL-143 was due to the presence of *HvFT3* gene. An additional QTL for TIL was identified in a very narrow region (97.18 to 98.23 cM) on chromosome 1H. This QTL was carried by two ILs S42IL-102 and S42IL-141 having overlapping wild introgression which helped us to narrow down the region to almost 1 cM. This QTL provide a straightforward opportunity to utilize these beneficial ILs for the positional cloning of underlying gene. The strongest QTL for RL was also identified on chromosome 1H between 199.04 to 205.07 cM that increased RL by 40.1% in S42IL-142. Hoffmann et al. [34] identified QTL on chromosome 1H for RDW in S42IL-102, for RL in S42IL-141 and S42IL-142 and for RL and RDW in S42IL-143 under two different levels of nitrogen application in hydroponic culture.

A small QTL region was linked to RDW, TIL and GH on chromosome 2H between 107.63 and 108.71 cM. We believe it may underlie a similar genetic component that promotes vegetative growth and subsequently influence root traits in S42IL-109. In wheat, Bai et al. [29] identified QTL for RDW on chromosome 2D which revealed microsynteny to barley chromosome 2H [48,49]. Champoux et al. [50] identified QTL for root thickness, root/shoot ratio and root dry weight per tiller below 30 cm in rice on chromosome 7 using recombinant inbred line (RIL) population. This chromosomal region also showed syntenic relationship with the barley chromosome 2H [50]. Naz et al. [13] detected major QTL for increasing vegetative growth and root trait variation on chromosome 5H which has also been confirmed in the present study. Although, QTL for GH and root traits were found together at some positions but RL, RDW, RV and TIL revealed unique QTL suggesting partial correlation of these traits. This fact led us to believe that the variation in root system variation in the S42IL population does not primarily depend on the GH. For instance, ILs S42IL-154 and S42IL-155 revealed a common introgression that carried only the QTL for RDW (*QRdw.S42IL.3H*) on chromosome 3H (64.85 to 65.96 cM). Likewise, unique QTL for RDW (*QRdw.S42IL.7H.b)*, RV (*QRv.S42IL.2H*) and RL (*QRl.S42IL.4Hb* and *QRl.S42IL.5H.a*) suggesting independent inheritance of these QTL in this population. Five QTL revealed the co-segregation of root traits RL, RDW and RV. These co-localization are in line with our phenotypic data that showed strong positive correlation among the root traits. Among these QTL, combined QTL for RDW and RL and RDW and RV were detected on chromosomes 4H and 6H in S42IL-116 and S42IL-129, respectively. Bai et al. [29] detected QTL for RDW, RV and RL on chromosome 4D of wheat. This QTL region may be syntenic to barley chromosome 4H [50,51] where we identified QTL for RDW and RL. Likewise, QTL for RDW and RV was found on chromosome 7H in S42IL-133. This region appears to contain semi-dwarf gene *brachytic 1* [51] that seems to link with a moderate increase of RDW (51.5%) and RV (49.8%) in S42IL-133 as compared to Scarlett. Taken together, these data reveal the identification of favorable QTL alleles of wild origin which accounted for improved root and related shoot traits in the Scarlett background. This fact revealed the utility of wild barley natural diversity and to bring back these valuable alleles into the elite breeding material. Hence, the fixation of these unique exotic QTL allele in the Scarlett background offers a direct opportunity to use them in barley breeding as well as to dissect underlying genes for basic research.

## Conclusions

Reversing the adverse effect of domestication and intensive selection is essential for sustainable crop production in agriculture. The present study was aimed to assess the utility of exotic alleles for the improvement of root and related shoot parameters under control and drought stress conditions. QTL mapping revealed 15 chromosomal regions where the introgression of exotic alleles resulted in improved trait-values. The favorable performance of these unique alleles in the cultivated background proves the utility of enriching wild gene pool in the elite breeding material. Therefore, ILs with agronomically beneficial QTL alleles have been achieved for a direct transfer of these valuable genetic resources to the modern cultivar. In addition, selected ILs were backcrossed with the recurrent parent Scarlett to refine the QTL regions as well as to develop high resolution mapping populations for the positional cloning of the underlying genes.

## Abbreviations

BBCH: Bundesanstalt, Bundessortenamt und CHemische Industrie (German scale to assess the development stage of plants)
BOPA: Barley oligo pool assays
SAS: Statistical analysis system
GLM: General linear model
